# Supplementary material for: Are carbon emissions trading and green financial instruments synergistic? -Comprehensive quantitative research based on content analysis
Source: PLoS One. 2024 Mar 7;19(3):e0298601. doi: 10.1371/journal.pone.0298601 (PMC10919601; doi:10.1371/journal.pone.0298601)
Supplement: S1 Data — (ZIP) [file pone.0298601.s001.zip › Data/地级市Z_F工作报告.zip!/║╙─╧/╜╣╫≈╩╨/└┤╫╘╣1⁄2╓┌║┼í╛┬φ┐╦╩2╛▌═°í┐_2011.docx]

各位代表：

　　现在，我代表市人民政府，向大会作政府工作报告，请予审议，并请各位政协委员提出意见。

　　一、 2010年及“十一五”工作回顾

　　2010年是“十一五”规划的最后一年，也是我市经济社会发展不平凡的一年。面对复杂形势和严峻挑战，在省委、省政府和市委的正确领导下，在市人大、市政协的监督支持下，市政府团结带领全市人民，深入贯彻落实科学发展观，强力实施工业强市战略，抢抓机遇，扎实工作，较好地完成了市十一届人大二次会议确定的各项目标任务，经济社会发展呈现出好的趋势、好的态势、好的气势。全年地区生产总值达到1247.6亿元，增长11.9%；地方财政总收入97.5亿元，增长12.9%，财政一般预算收入63.3亿元，增长16.4%，其中市本级收入17.8亿元，增长19.2%，增收2.87亿元；规模以上工业增加值达到706.5亿元，增长17.3%；全社会固定资产投资970.8亿元，增长20.8%，其中城镇固定资产投资841亿元，增长21%；社会消费品零售总额321.8亿元，增长18.3%。

　　（一）大力推进项目建设，发展基础更加坚实。全市新开工项目1641个，完成投资538.7亿元。加快重点项目建设。深入开展“项目服务年”、“企业服务年”活动，一批重大项目实现突破性进展，140个市重点项目完成投资226.3亿元。中轴集团年产1万辆专用汽车、昊华宇航年产24万吨离子膜烧碱和20万吨聚氯乙烯、多氟多年产200吨六氟磷酸锂、220千伏宁郭输变电工程、河南工程技术学校新校区等项目建成投用，焦煤集团开元化工年产15万吨环氧氯丙烷和8万吨环氧树脂、新乡至月山铁路增建第二双线、郑焦城际铁路云台山支线、职教园区、国际太极拳文化交流中心暨全民健身中心等项目开工建设，郑焦城际铁路、龙源电厂2×60万千瓦机组、人民路东西延伸、中国兵器工业焦作光电产业园、风神轮胎年产1000万套高性能乘用子午胎（一期）等项目顺利推进。有效破解资金难题。多氟多、中内配、新开源3家企业在A股上市，大通化工在澳大利亚证券交易所上市，全市上市企业总数居全省第二位；淼雨饮品等6家企业在天津股权交易所挂牌。交通银行、光大银行、广发银行落户焦作，武陟射阳村镇银行开业运营，沁阳江南村镇银行成功签约。强力推动银企对接合作，积极争取上级资金支持，全年融资总额133亿元，上级转移支付资金52.76亿元。筑牢项目承载平台。焦作新区各项工作全面展开，空间发展战略规划初步方案通过专家评审。9个省级产业集聚区建成面积50.9平方公里，入驻企业1150家，实施亿元以上工业项目127个；完成固定资产投资330亿元，规模以上工业主营业务收入、利润、利税分别实现905亿元、84亿元、114亿元，增长31%、18%、15%，产业集聚区综合排名位居全省前列。

　　（二）积极优化产业结构，发展方式加快转变。做大做强工业。六大战略支撑产业和四大新兴产业分别实现规模以上工业增加值399.8亿元、26.5亿元，增长17.1%、30.9%。出台了加快中心城区发展的意见，以及加快中心城区工业、服务业、金融业、旅游业发展的配套意见，明确了提升传统产业的方向，制定了壮大新兴产业的措施，启动了首批十大工业特色产业园区建设。与中国化工、台盟中央签订了战略合作协议，促成了科艺纺织与恒祥纺织集团、联盟卫材与修正药业、鑫安药业与北京福瑞堂、坚固水泥与河南博源等企业战略重组。推动老城区工业企业搬迁，中轴集团东风机器厂等5家首批搬迁企业开工建设。新增国家级企业技术中心1家、省级工程技术研究中心4家、省级企业技术中心7家；全市高新技术产业增加值达到161.3亿元，增长20%。夯实农业基础。全年粮食总产199.4万吨，增产1.6万吨，连续７年实现丰产丰收。肉、蛋、奶产量分别达到19.2万吨、28.2万吨、22万吨，完成营造林13万亩，发展节水灌溉8万亩。新增“一村一品”专业村20个，总数达到245个。新增农民专业合作社594个，总数达到2300个。天香面业成为全省唯一增加的农业产业化国家重点龙头企业。全市规模以上农产品加工企业实现销售收入283.3亿元、利润13.7亿元，分别增长14.3%、8.1%，被命名为全国优质小麦加工示范基地和全国农产品加工创业示范基地。农机总量64万台，亩均动力达到1.43千瓦，位居全省第一；粮食生产机械化水平达到86%，成为首家地市级全国农业机械化示范区。繁荣第三产业。云台山国际旅游节升格为国家旅游局、河南省人民政府主办，在首届中国旅游节庆品牌峰会上荣获“2010年度最佳旅游节庆奖”。神农山成功创建AAAAA级景区，圆融寺、嘉应观成功创建AAAA级景区。全年接待游客1959万人次、门票收入6亿元、旅游综合收入147.5亿元，分别增长15.2%、15.5%、15.2%。万村千乡市场工程、新农村现代流通服务网络工程、家电下乡工程扎实推进，建成农家店198家，总数达到1614家，行政村覆盖率达到96.8%。物流、保险、电子商务等现代服务业快速发展。加快城市转型，编制了《焦作市资源枯竭城市转型规划》，顺利通过国家资源枯竭城市转型中期评估，未来５年将持续得到国家支持。

　　（三）加强基础设施建设，城乡环境逐步改善。南水北调工程建设顺利推进，总干渠89.1万平方米安置房交付使用，2.7万名群众得到妥善安置，绿化带46万平方米安置房主体基本完工，荣获“全国南水北调工程征迁工作先进集体”称号，是全国唯一受表彰的地级市。进一步完善城市功能，实施了解放东路、建设西路、文昌路、陶瓷路大修工程，完成了丰收路改造、迎宾路绿化工程，新增供水管网42公里、供热面积50万平方米。数字城管监控指挥平台与部分县市区联网运行，城市管理水平进一步提升。强化国土资源管理，保障建设用地1.7万亩，被评为“全国双保工程先进单位”（保障经济发展、保护耕地红线）。大力发展交通公路事业，桃花峪黄河大桥、林邓线一级公路建设加快，焦桐高速、云郑高速、焦作黄河大桥前期工作进展顺利。加强新农村建设，解决了18.7万农民和1.65万农村学校师生饮水安全问题，搬迁了4个贫困自然村，解决了1.2万贫困人口温饱问题，建成农村住宅楼288栋111.6万平方米，新增户用沼气1万户，治理水土流失面积40平方公里，新建改建农村公路295.8公里，完成9座病险水库除险加固任务，沁河下游治理项目列入国家黄委会“十二五”规划。

　　（四）继续深化改革开放，发展活力不断增强。完成了市县政府机构改革，实施了警务体制机制改革，推进了轮胎集团、万方集团、化电集团改革工作；加快农村信用联社体制改革，修武、孟州农村信用联社分别组建农村商业银行，市商业银行在沁阳设立了支行；借鉴重庆经验，规范完善了五大投融资公司。持续开展大招商活动，成功举办了珠三角经贸合作和旅游推介、长三角经贸洽谈、深港澳地区闽籍企业家招商等一系列重大招商活动，全市新引进市外境内项目864个，总投资681.4亿元；实际利用外资2.88亿美元，增长45.6%；全年实现进出口总值17.58亿美元，增长63%，其中出口实现10.7亿美元，增长53.9%。焦作海关筹建进展顺利。与波兰共和国卢布林市签订了国际友好城市协议，组团访问了韩国首尔、忠州等城市，对外交流合作进一步加强。

　　（五）高度重视民生改善，社会大局和谐稳定。投资5.15亿元，办好十项民生工程。实施中小学校舍安全工程，112个项目全部竣工，建成面积16万平方米；重视义务教育均衡发展，人民中路学校建成投用，积极推进职业教育资源整合，支持高等教育发展，教育质量不断提高。改造了14个中心乡镇卫生院、26个城市社区卫生服务中心，新区中医院建设进展顺利，新农合参合率达到97.69%。人口计生工作扎实推进，被确定为“全国婚育新风进万家活动第四阶段示范市”。积极参加第十一届省运会，总成绩位居全省第三，成功申办第十二届省运会。全市城镇新增就业8.4万人，下岗失业人员再就业3.2万人。圆满完成城镇居民基本医疗保险和农村新型养老保险国家试点任务。市直城镇职工基本医疗保险、新农合最高支付限额均提高到6万元，城区低保标准提高到210元，五保对象集中供养率达到68%。免费为2833例白内障患者实施复明手术，荣获“全国白内障无障碍市”称号。开工建设廉租住房22万平方米，累计竣工5576套27.9万平方米；棚户区改造21个项目全面启动。完成了城区5座危桥加固改造工程，建设改造燃气管网59.6公里，疏通了普济路供热管网，新购公交车30台。深入推进环境污染整治，市区环境空气优良天数达到314天。治理整顿煤球市场。实施“网络问政”，重视社情民意，解决了一批事关人民群众切身利益的实际问题。

　　推进“平安焦作”创建活动，加强社会治安防控体系建设，人民群众安全感和满意度进一步提升，被评为“平安建设先进省辖市”；深入开展矛盾纠纷排查化解，信访形势保持稳定；严格落实安全生产责任制，安全生产形势平稳；深入创建“食品安全示范市”，完善了检测体系和风险评估体系。人民防空工作扎实推进，应急管理体系建设进一步加强。民族关系团结和睦，民族经济进一步发展。积极推进军民融合式发展，军政军民关系和谐融洽。驻焦部队、武警官兵和广大民兵预备役人员积极支持参与地方经济社会发展，在防汛抗旱、抢险救灾和维护社会稳定中作出了重要贡献。

　　（六）注重政府自身建设，行政效能明显提升。出台了《转作风提效率促落实若干规定》，明确了作风建设六条要求。市政府年度主要工作目标任务以及完成情况向全社会公示。清理行政审批事项588项，取消行政审批事项318项，电子监察系统试运行。组建了市公共资源交易中心，完成交易事项238个，交易总额22亿元。落实政府性投资建设项目“十个百分之百”管理，审核资金38.9亿元，审减资金5.7亿元，审减率14.5%。

　　（七）认真对待代表委员意见，切实解决热点难点问题。对人大代表、政协委员提出的意见和建议，市政府高度重视，多次召开会议专题研究，实行分管市长负责制，明确办理责任，将办理工作纳入市政府目标考核体系，制定考核细则，完善办理机制，加强与人大代表、政协委员的沟通，多种形式征求意见，重点办理、跟踪办理、延伸办理，154件市人大代表建议和538件市政协委员提案全部办结。建立了市政府与市人大代表、政协委员网络联系平台，主动及时通报政府工作情况，人大代表、政协委员提出的保障外来务工女性权益等13条建议得到较好办理。

　　第六次人口普查工作进展顺利，审计、工商、质监、检验检疫、地震、气象、广电、档案、史志、宗教等工作都取得了新的成绩。

　　各位代表，2010年工作目标的实现，标志着我市“十一五”主要任务胜利完成。“十一五”期间，我们经历了国际金融危机的严重冲击，经历了经济环境复杂多变的严峻挑战，经历了电力供应紧张、天气持续干旱的重大考验。在挑战和考验面前，我们科学决策、积极应对，全市人民团结一心、克难攻坚，保持了经济社会平稳较快发展的态势，夯实了跨越式发展的基础，推动经济社会进入了蓄势勃发的新阶段。

　　这五年是经济快速发展、综合实力迈上新台阶的五年。地区生产总值年均增速13.6%，经济总量由559.9亿元增加到1247.6亿元，地方财政总收入由54.1亿元增加到97.5亿元，规模以上工业增加值由252亿元增加到706.5亿元。

　　这五年是加快发展方式转变、产业结构优化升级的五年。坚持走新型工业化道路，六大战略支撑产业发展壮大，四大新兴产业初具规模，主营业务收入超百亿元企业1家、超50亿元企业4家、超亿元企业520家。科技创新步伐加快，被评为“全国科技进步先进市”。现代农业、特色农业健康发展，农产品加工企业达到673家。旅游业发展势头强劲，创建AAAAA级景区2家、AAAA级景区3家，荣获“太极圣地”、“中国最佳休闲旅游城市”称号。

　　这五年是城乡建设不断加快、城市形象显著提升的五年。市区建成区面积由75平方公里增加到95平方公里，城镇化率预计达到48.4%，5年提高8.4个百分点。新建改建农村公路4675公里，公路通车总里程7316公里，公路密度每百平方公里达到180公里，是全国平均水平的4.5倍，市区和各县市垃圾卫生填埋场、污水处理厂全部建成投用。人均公共绿地面积达到8.9平方米，荣获“国家园林城市”称号。

　　这五年是各项改革深入推进、对外开放不断扩大的五年。政府机构、公共财政、国有企业、集体林权、金融、投融资体制等改革稳步推进。与中国铝业、中国化工、中国兵器工业集团、厦工股份、河南煤化等一批央企省企和行业龙头企业深化合作，5年引进亿元以上项目354个，出口总额实现33.5亿美元，与156个国家和地区建立了经贸往来关系。

　　这五年是社会事业全面进步、人民得到更多实惠的五年。坚持每年办好十项民生工程，5年累计投入资金28.5亿元。城镇新增就业41万人次，农村劳动力转移就业64.2万人。城乡办学条件得到改善，全部拆除中小学D级危房，改造B、C级危房，新建了人民中学等一批学校。全市1985个行政村和社区全部建成群众体育健身工程。城镇居民基本医疗保险全面建立，新农合参保人数244万人。实现8.2万贫困人口脱贫，解决了71.25万人的饮水安全问题。城镇居民人均可支配收入达到15781元，农民人均纯收入达到7512元，分别是“十五”末的1.9倍、2倍。

　　各位代表，“十一五”成绩的取得，是省委、省政府和市委正确领导的结果，是市人大、市政协积极支持的结果，是全市人民共同努力、艰苦奋斗的结果，凝聚着各位代表、各位委员的智慧和力量，饱含着广大干部群众的心血和汗水。在此，我代表市人民政府，向工作在各条战线的工人、农民、知识分子、干部、驻焦部队官兵、武警官兵、公安民警，向各民主党派、无党派人士、各人民团体，向人大代表、政协委员，向所有关心支持焦作改革发展的各界人士，致以崇高的敬意和衷心的感谢！

　　各位代表，回顾五年来的工作，历程很不平凡，成绩来之不易，经验弥足珍贵。我们深刻体会到，要始终以科学发展观为统领，不断提高科学发展的能力，有“六个坚定不移”需要长期坚持并努力做好：一是坚定不移走转型升级之路，加快经济发展方式转变，在发展中转变，在发展中调整，在发展中提升，在发展中增效；二是坚定不移走工业强市之路，始终坚持工业强市战略，壮大工业经济规模和实力；三是坚定不移走项目带动之路，集中精力谋划、研究、推进重大项目建设，切实增强发展后劲；四是坚定不移走改革开放之路，深入推进体制机制改革创新，提高对外开放的层次和水平，增强发展的动力和活力；五是坚定不移走科技创新之路，加强自主创新体系建设，培育高新技术企业和创新型企业，增强科技支撑能力；六是坚定不移走以人为本之路，把保障改善民生作为根本出发点和落脚点，增加民生投入，着力解决就学、就医、就业、住房和社会保障等关系人民群众切身利益的问题，发展成果让人民群众共享，依靠人民群众共建美好家园。

　　各位代表，在肯定成绩的同时，我们也清醒地看到发展中存在的矛盾和问题：一是经济发展方式粗放的状况尚未根本扭转，长期积累的结构性矛盾较为突出，工业产品多数集中于产业链的前端和价值链的低端，服务业发展相对滞后；二是自主创新能力不强，科技创新人才不足，高新技术产业规模不大；三是资金保障、环境容量、土地供应等要素制约日益显现，保持经济平稳较快发展的压力加大；四是水、电、气、路、桥等城镇基础设施建设的任务还很重，中心城区出现交通拥堵，城市管理水平有待进一步提高；五是公共服务、民生改善与广大群众的期盼仍有较大差距，山区丘陵区群众吃水难问题还没有得到根本解决；六是一些干部领导方式和工作作风还不适应科学发展的要求，服务意识不强、工作效率不高、落实不到位，政府自身建设尚需进一步加强等。对这些问题，我们将采取有效措施，认真加以解决。

　　二、“十二五”时期的指导思想和发展目标

　　“十二五”时期是我市加快科学发展、全面建设小康社会的关键时期，是深化改革开放、加快转变经济发展方式的攻坚时期，也是必须紧紧抓住并实现快速发展的重要战略机遇期。省委八届十一次全会审议通过的中原经济区建设纲要，对建设中原经济区作了全面部署，将引领河南步入新的发展阶段。焦作处于中原经济区核心区，在中原经济区建设的伟大实践中，我们要凝聚全市人民的智慧和力量，抢抓机遇，加倍努力，扎实苦干，奋发有为，实现经济社会跨越式发展。

　　“十二五”时期经济社会发展的指导思想是：以邓小平理论和“三个代表”重要思想为指导，深入贯彻落实科学发展观，按照“四个重在”的实践要领，紧紧抓住中原经济区建设重大机遇，以科学发展为主题，以加快转变经济发展方式为主线，以富民强市为中心任务，强力实施工业强市战略，以新型城镇化带动工业化、农业现代化“三化”协调发展，不断增强产业集聚能力、自主创新能力、城乡统筹发展能力和区域竞争能力，奋力走在中原经济区前列，建设更具活力的新型城市。

　　“十二五”时期经济社会发展的目标和愿景是：

　　——综合实力显著增强。地区生产总值增速高于全省平均水平，财政一般预算收入与地区生产总值年均增速同步，人均主要经济指标高于中原经济区核心区平均水平，力争三年一大步、五年翻一番，综合实力和区域竞争力位居中原经济区前列。

　　——发展方式显著转变。基本形成以高新技术企业和特色基地为支撑的现代工业体系，农业现代化水平明显提高，现代服务业快速发展，形成一批拥有自主知识产权和知名品牌、国际竞争力较强的优势产业和企业群体。

　　——城乡面貌显著改善。建成集生态绿化、观光旅游、休闲娱乐于一体的南水北调景观带，城市水系基本形成，空气质量明显优化，农村环境明显改观，交通路网日趋完善，城市品位有较大幅度提升，环境优美、生态和谐的山水园林城市初具雏形。

　　——生活水平显著提高。基本公共服务体系覆盖城乡，教育、文化、卫生、体育等公共设施布局更加优化，人民群众生活更加殷实，幸福指数进一步提高。五年后，一个充满生机、更具活力的新焦作将展现在我们面前。

　　《焦作市国民经济和社会发展第十二个五年规划纲要（草案）》及说明已印发各位代表，请予审议。

　　三、2011年工作的主要目标和任务

　　2011年是“十二五”开局之年。总体上看，我市今年面临的发展形势依然复杂。国际金融危机影响仍然存在，世界经济不确定因素很多，国家实施宏观调控，发展要素供给趋紧。与此同时，世界经济继续复苏，我国经济发展趋势持续向好，中原经济区建设上升至国家战略层面，我们面临更多的发展机遇。压力和动力同在，机遇和挑战并存。我们将坚定信心，抢抓机遇，发挥优势，加快发展，确保“十二五”开好局、起好步。

　　今年经济社会发展主要预期目标是：地区生产总值增长12%；地方财政一般预算收入增长12%；规模以上工业增加值增长16%；全社会固定资产投资增长20%，其中城镇投资增长21%；社会消费品零售总额增长18.5%；外贸出口增长21%；实际利用外商直接投资增长22%；居民消费价格涨幅控制在4%左右；人口自然增长率控制在6.5％。以内；城镇登记失业率控制在4.5%以内；城镇居民人均可支配收入增长10%，农民人均纯收入增长10%；节能减排完成省定目标。

　　实现上述目标，工作中必须把握好以下原则：一是着力融入大局。主动呼应、主动对接、主动融入中原经济区建设，以更加开放的思维、更加前瞻的眼光，谋划发展、加快发展。二是着力结构调整。突出产业结构调整，推动产业优化升级，促进经济社会又好又快发展。三是着力项目建设。强化项目意识，大手笔谋划项目，大力度推进项目，进一步夯实发展基础。四是着力统筹运作。在经济发展和社会建设、推进“三化”进程上统筹运作，探索走出一条不以牺牲农业和粮食、生态和环境为代价的“三化”协调发展的路子。五是着力改革开放。靠改革创新破解深层次矛盾，靠扩大开放实现借势发展。六是着力民生民心。把群众呼声作为第一信号，把群众需求作为第一选择，把群众满意作为第一标准，尽心竭力为人民群众办好事办实事，让改革发展成果更多更好地惠及人民群众。

　　今年重点抓好八个方面工作：

　　（一）以项目建设为抓手，全力保持经济平稳较快发展。始终坚持项目带动，一切工作依靠项目开展，一切资源向项目倾斜，用项目支撑焦作发展。全年计划实施5000万元以上项目550个，总投资1900亿元，今年计划完成投资600亿元。其中，工业项目330个，总投资1000亿元，今年计划完成投资350亿元。竣工投产一批项目。风神轮胎年产1000万套高性能乘用子午胎（一期）、河南煤化焦煤集团年产20万吨离子膜烧碱、焦煤集团年产1000吨多晶硅、中国兵器工业焦作光电产业园一期工程等项目竣工投产。加速推进一批项目。加快焦煤集团开元化工年产15万吨环氧氯丙烷和8万吨环氧树脂、龙源电厂2×60万千瓦机组、河南思可达年产700万平方米太阳能封装玻璃等项目建设。开工建设一批项目。焦作电厂2×66万千瓦机组环保迁建、焦作东区2×30万千瓦热电机组、龙星化工年产14万吨炭黑装置和尾气综合利用、多氟多年产1亿安时动力锂电池、焦作万方年产10万吨铝型材加工、焦作海关等项目开工建设。前期运作一批项目。深圳鼎利源资产管理公司低碳产业园、修正中原焦作产业园、丹河电厂2×100万千瓦机组易地迁建、江苏兴达年产20万吨钢帘线、徽铝铝业年产10万吨高档铝型材等项目争取早日落地。认真谋划一批项目。围绕优势产业拉长链条，围绕对接央企省企，围绕基础设施建设，围绕城乡统筹发展，围绕保障改善民生，谋划一批带动作用强、科技含量高、经济社会效益好的重大项目，为加快发展提供有力支撑。

　　（二）以结构优化为重点，加快构建现代工业体系。改造提升传统产业，培育发展新兴产业，延伸产业链条，壮大经济总量，提高工业经济的质量和效益。做强十大产业。围绕产业链上下游延伸，装备工业重点发展工程机械、造纸机械等，汽车及零部件产业向系统化、模块化方向发展，铝工业向铝型材等精深加工方向发展，煤盐联合化工产业开发多晶硅等高附加值产品，能源工业加快推进电力项目建设，食品工业积极发展精深加工。加大扶持力度，培育壮大生物制药、新材料、新能源、节能环保四大新兴产业。2011年，六大战略支撑产业、四大新兴产业实现销售收入突破2000亿元，占全市工业的比重达到65%。建设十大园区。围绕重点骨干企业，加快建设焦作（焦煤集团）新材料产业园、风神轮胎产业园等中心城区首批十大工业特色产业园区，打造一批产业关联度强、科技含量高、市场前景好的产业集群。2011年十大园区销售收入突破200亿元。抓好十大平台。加快焦作新区和9个省级产业集聚区基础设施建设，加大项目引进力度，2011年产业集聚区基础设施投资达到50亿元，工业投资达到300亿元，主营业务收入达到1100亿元。搬迁10家企业。力争老城区10家工业企业成功搬迁，真正搬出规模和效益，搬出发展的新天地。

　　（三）以发展现代农业为方向，促进农业增效农民增收。加大财政投入力度，认真落实强农惠农政策，加快传统农业向现代农业转变。深入开展粮食高产创建活动，实施高产创建整县制推进试点，高标准建设一批万亩示范片、千亩示范方、百亩攻关田，进一步提高粮食生产能力。加快小麦种子、“四大怀药”、食用菌、无公害蔬菜、林果与畜牧六大特色产业发展，加快发展现代畜牧业，建成36个标准化规模养殖场，探索发展观光农业。强力实施“百户龙头企业培育工程”，大力发展农产品深加工，加快农业产业化，做大做强一批国家级、省级农业产业化重点龙头企业。加快农民专业合作社发展，新增国家级示范社1家、省级示范社5家、市级示范社20家。巩固和深化全国农机化示范区创建成果，新增农机具4500台，进一步提高粮食生产机械化水平。抢抓中央加快水利改革发展的重大机遇，加强农田水利建设，实施节水灌溉、病险水库除险加固、中小河道治理等水利工程，加快林业生态市建设，推进沁河、新河、大沙河治理工程，建设林区生态保护型人工增雨示范基地，改善农业综合生产条件。加快推进农村饮水安全工程建设，3年内平原地区全部用上自来水，3年内解决山区丘陵区吃水问题，让人民群众彻底告别吃水难，吃上放心水，吃上安全水。

　　（四）以文化旅游业为龙头，提升服务业发展水平。围绕打造国际知名旅游城市，抓好云台山文化休闲区、神农山文化旅游综合服务区、陈家沟太极拳文化旅游园区、圆融寺文化旅游区、影视文化旅游区、黄河文化旅游区六大文化旅游园区建设，推进青天河、圆融寺和嘉应观争创AAAAA级景区。加快博晋路至月山寺、获轵线大修等旅游道路建设。办好第六届中国焦作国际太极拳交流大赛，筹划拍摄一部高水平的太极拳电视剧精品，加快太极拳申报世界非物质文化遗产工作，进一步扩大太极拳影响，做大太极文化产业。支持云台山旅游股份有限公司加快上市。围绕“吃、住、行、游、购、娱”，拉长旅游产业链条，加快中心城区旅游业发展。加强煤文化遗迹保护开发，筹建焦作煤文化博物馆。培育挖掘焦作精神，丰富城市文化内涵。

　　大力引进域外金融机构，全年引进1~2家股份制商业银行，力争设立2~3家村镇银行；支持市商业银行引进战略合作者，向县市拓展营业网点；加快企业上市步伐，力争3家企业成功上市；加快五大投融资公司发展，推进农村信用联社改制工作，加强金融生态环境建设，推动证券、保险、基金、信托、担保、租赁等行业创新发展。引进大型商贸龙头企业，提升中心城区商贸业档次；积极推进万村千乡市场工程、新农村现代流通服务网络工程、家电下乡工程、社区便民服务工程、放心早餐工程建设，发展提升电子商务、网络购物等新型业态，满足群众多样化消费需求；扶持现代物流业发展，实施《焦作市现代物流业发展规划》，积极推进焦东综合物流园区、宏达物流园区等项目；整顿规范市场秩序，营造良好消费环境，进一步繁荣市场、扩大内需。

　　（五）以新型城镇化为引领，统筹城乡发展。坚持以产兴城、以城带乡，以新型城镇化带动“三化”协调发展，构建中心城区—县城—小城镇—农村新型社区相互衔接的城乡统筹发展格局。加快中心城区发展。全力支持焦作新区建设，加快区划调整，理顺体制机制；改革创新服务平台，建设高效、便捷、为民的公共资源交易平台；强力推进一批工业、基础设施、社会事业和民生项目，尽快出亮点、见成效。力争经过几年努力，把焦作新区建成焦作市“三化”协调发展先导区、老工业基地振兴和资源型城市转型示范区、现代化复合型功能区、城乡统筹发展先行区和对外开放示范区，成为焦作城市发展的一个亮点，走在全省新区建设的前列。科学编制规划，完成焦作市总体城市设计及重要地段城市规划设计；严格规划管理，坚决维护规划的法定性、权威性和严肃性。积极发展城市公交，加快公共停车场规划建设，改善群众出行条件。加快中心城区供水、供气、供暖等基础设施建设，启动污水处理厂升级改造工程，改造老城区燃气管网60公里、供水管网28公里，新增集中供热面积40万平方米，作好城市水系规划建设，抓好太行山绿化、西部万亩森林生态园等工程建设。加快旧城改造、城中村改造，引进开发现代城市综合体，规划建设城市标志性景观，进一步提升城市品位。全面推进南水北调城区段二期征迁工作，完成绿化带安置房建设，开工建设绿化带和11座跨渠桥梁、3条市政管廊工程，积极推进5个配套水厂前期工作。开展城乡环境卫生整洁行动，推进数字城管建设上档升级，进一步提高城市管理水平，营造卫生有序的城市环境。推进TD无线城市建设，加快城市信息化进程。加快新农村建设。加快推进城镇化进程，统筹城乡发展。每个县市建好一个产业集聚区，着力培育一个以上销售收入超百亿元的特色主导产业和一个人口规模超20万的城市。因地制宜发展小城镇，支持基础条件好的中心镇，通过加快专业园区建设，逐步发展成为小城市。积极推进新型农村社区建设，抓好114个省市县三级示范村，实施新一轮农村扶贫开发，完成省定贫困村整村推进任务，新增户用沼气1万户，整治村容村貌，加强农村环境保护，控制农村面源污染，改善农民生产生活条件，逐步实现城乡基本公共服务均等化。加快交通体系建设。加快郑焦城际铁路及云台山支线、新乡至月山铁路增建第二双线建设，积极推进焦作至济源、洛阳和焦作至晋城城际铁路项目；开工建设云郑高速公路、焦桐高速公路温县至巩义段项目；加快桃花峪黄河大桥建设，强力推进焦作黄河大桥、孟州黄河大桥项目前期工作；开工建设迎宾路向南延伸工程，实现与连霍高速无缝对接，推进三条拟升国道和六条拟升省道项目前期工作；充分发挥五大投融资公司的作用，启动环城快速通道建设，实施南水北调两侧绿化带道路和建设路、中原路等建设，实施解放西路、南通路等大修工程，建成人民路东西延伸工程、林邓线一级公路。今年谋划实施城市道路建设工程29个，总投资47.5亿元，是“十一五”期间投资的3.3倍；新改建农村公路200公里。通过不同层次的交通建设，积极构建铁路、高速公路、黄河大桥、干线公路、城市道路、农村公路协调发展的现代交通网络体系，拉近与郑州等周边城市的距离，打造客流大通道，构建交通大优势。

　　（六）以科技创新为支撑，加快两型社会建设。培育创新主体，打造创新载体，加强自主创新体系建设。强化企业主体意识，引导企业加大技术改造和产品研发力度，开展产学研合作，争取新增省级企业技术中心5家、工程技术研究中心3家。争取新增高新技术企业3家，高新技术产业增加值增长20%。实施《焦作汽车零部件特色产业升级规划》，推动国家火炬计划焦作汽车零部件特色产业基地建设。围绕“四大怀药”科技开发，争创国家中草药保健品基地。强化品牌建设，继续实施市长质量奖，引导企业加强质量管理和标准化体系建设，争创一批知名商标、名优品牌。加强人才队伍建设，培育创新型人才，为经济社会快速发展提供智力支持。

　　加快资源节约型和环境友好型社会建设。认真落实节能减排目标责任制，抓好高耗能行业技术改造，加快淘汰落后产能，实施东方金铅系统节能改造等9个项目建设；开展全民节能行动，建设绿色建筑和低碳社区。严格土地管理，推进矿山地质环境恢复治理项目建设，实现保障经济发展、保护耕地红线“双保”目标。建设多尔克司乳业等一批循环经济示范基地。实施“1790污染整治工程”，加强重点流域、重点区域、重点行业环境污染综合治理，深入推进排污权交易试点工作。加快市、县第二轮城镇污水处理厂建设，积极推动中水利用，建立区域大气污染联防联控机制，进一步提升生态环境质量。

　　（七）以改革开放为动力，增强经济社会发展活力。深入推进财政体制改革、警务体制机制改革、投融资体制改革、医药卫生体制改革、农村土地流转、集体林权制度改革等工作；推进市属企业脱钩改制工作，深化轮胎集团、万方集团、化电集团等国有企业改革发展。瞄准世界500强、国内500强、央企省企和行业龙头企业，力争引来一批大项目、好项目，培育发展产业集群。充分利用国家促进东部地区加工贸易向中部转移的政策机遇，积极承接加工贸易产业转移。鼓励支持城区发展“飞地工业”。加强与台资企业、闽籍企业沟通对接，抓好台湾工业园、台湾农民创业园建设。完善信用体系，打造信用焦作。充分利用国际国内两个市场、两种资源，调整出口产品结构，积极开拓海外市场，扩大企业出口规模，进一步发展外向型经济。加强对外联络，实现对外交流合作新发展。

　　（八）以保障改善民生为根本，积极推进和谐社会建设。把保障改善民生放在更加突出的位置，继续办好十项民生工程，着力解决一批民生问题，努力让人民群众生活得安心、舒心。实施中长期教育改革和发展规划纲要，启动学前教育三年行动计划，做好新一轮中小学布局调整工作，完成100个校舍安全工程，加快职教园区建设，发展高等教育，支持升本工作；抓好市委党校扩建工程，建设高标准的干部培训教育基地。启动青少年活动中心和妇女儿童活动中心项目前期工作，继续实施文化信息资源共享、农家书屋、农村数字电影放映等文化惠民工程，完成乡镇综合文化站建设任务。建成新区中医院，完成3所县级医院建设改造任务，建成15个城市社区卫生服务中心，完善农村医疗卫生服务网络。加强人口计生工作，稳定低生育水平，统筹解决好人口问题。以承办第十二届省运会为契机，积极推进国际太极拳文化交流中心暨全民健身中心建设。强化就业指导，优化就业服务，拓宽就业渠道，搭建就业平台，扩大对零就业家庭成员和就业困难人员的就业援助，全年新增就业6.2万人。加快建设覆盖城乡居民的社会保障体系，认真解决失地农民生活保障问题，大力发展养老服务业，推进养老服务设施建设，做好新型农村社会养老保险试点工作；建成投用流浪未成年人救助中心；加强残疾人帮扶工作，做好残疾人就业培训和服务工作。开工建设廉租住房1220套、公共租赁住房3000套、棚户区改造8900套，进一步改善低收入群体居住条件。抓好“菜篮子”工程，保障市场供应；严格价格监管，严厉打击哄抬物价等不法行为；完善最低生活保障、失业保险标准与物价上涨挂钩的联动机制，保障低收入群体基本生活。加快老区建设，切实改善老区群众生产生活条件。深化和谐社区示范创建活动，加强和改进社区服务工作。启动“六五”普法规划，深化“平安焦作”创建，完善社会治安防控体系建设，推进社会管理创新。健全信访工作机制，畅通群众利益诉求渠道。严格落实安全生产责任制，坚决遏制重特大事故发生。开展“食品安全整顿年”活动，加强食品安全评估工作，让人民群众吃上健康放心食品。加强应急工作，提高突发公共事件处置能力。发展人民防空事业，推进防空防灾一体化建设。大力加强国防教育，积极支持驻焦部队和民兵预备役建设；创新开展双拥共建工作，巩固发展军政军民团结，不断提高国防后备力量建设和军地融合发展水平。全面落实党的民族宗教政策，巩固发展平等、团结、互助的民族关系，依法管理宗教事务，充分发挥民族和宗教界人士在促进经济社会发展中的积极作用。继续做好审计、统计、检验检疫、地震、广电、档案、史志等工作。

　　四、切实加强政府自身建设

　　发展的重任、人民的意愿，对政府自身建设提出了新的更高的要求。我们必须用领导方式转变加快发展方式转变，坚持“三具两基一抓手”，深入推进“两转两提”，建设人民满意的政府。

　　（一）勇于担当。为了焦作发展和人民福祉，要始终保持强烈的事业心和责任感，敢于负责，勇于负责，面对矛盾不回避，遇到难题不推诿，雷厉风行不拖拉。多做打基础、管长远的事，不搞形象工程，不搞短期行为。加大对基层的支持力度，激发全市上下干事创业的积极性和创造性。牢固树立为民意识，对人民群众的承诺，必须不折不扣落实到位；对人民群众的诉求，必须严格实行首问负责制；对人民群众的困难，必须真心实意帮助解决；对损害人民群众利益的行为，必须严肃查处、坚决纠正。

　　（二）加强学习。各级领导干部要勤于学习、善于学习，着力建设学习型政府、学习型机关。深入学习“何平九论”，加快转变领导方式。注重学以致用，紧密结合工作实际，把学习的成果转化为谋划工作的思路、统筹工作的本领、促进工作的措施，提高驾驭市场经济、应对复杂局面、推动科学发展的能力和水平。

　　（三）依法行政。深入推进法治型政府（机关）建设，严格按照法定权限和程序行使权力、履行职责。自觉接受人大及其常委会的法律监督、工作监督和政协的民主监督，对人大代表建议、政协委员提案办理的满意度情况进行考核。认真听取各民主党派、工商联和无党派人士的意见，充分发挥工会、共青团、妇联等人民团体的作用。加强政务公开，及时向社会通报政府工作进展情况，继续坚持年初公布政府年度目标任务、年终公布完成情况。办好市政府与市人大代表、政协委员网络联系平台，让沟通更加便捷。

　　（四）注重运作。科学运作是提高工作成效的重要保证。要持续行之有效的做法，不动摇、不懈怠、不折腾。遵循客观规律，找准贯彻落实上级决策部署的切入点、结合点、着力点，创造性地开展工作。讲究运作、善于运作、精心运作，把各项工作落实到项目上，落实到实质性操作层面上，以运作促工作，以运作促发展。

　　（五）求实求效。坚持求真务实，认真调查研究，吃透基层情况，科学民主决策，使工作和思路切合当地实际、符合群众意愿。强力推动工作落实，坚持实行工作台账制，细化责任，跟踪问效；坚持实行限时办结制，当日事当日毕，定下的事抓紧办、限时办，提高行政效能和工作效率；坚持实行绩效评估制，完善评估体系，加强绩效评估；坚持实行行政问责制，严肃整治不作为、慢作为、乱作为，确保各项决策部署落到实处、见到实效。

　　（六）廉洁从政。认真落实党风廉政建设责任制，统筹推进教育、制度、监督、改革、惩治等工作。狠抓廉政风险防控机制建设，加强对权力运行的全程监控，坚持政府性投资建设项目“十个百分之百”管理，让权力在阳光下运行。严格控制行政开支，公务接待、因公出国（境）、公务用车购置及运行维护三项经费支出预算原则上实行零增长。坚持“严管是最大的爱护”，努力建设一支清正廉洁、干净干事的公务员队伍。

　　各位代表，站在新的起点，实现新的跨越，是时代赋予我们的光荣使命和神圣职责。让我们在市委的坚强领导下，凝聚全市人民的智慧和力量，万众一心，奋发图强，锐意进取，扎实工作，为奋力走在中原经济区前列、建设更具活力的新型城市、谱写“十二五”辉煌灿烂的新篇章而努力奋斗！
